# Lean-Based Approach to Improve Emergency Department Throughput

**DOI:** 10.7759/cureus.69591

**Published:** 2024-09-17

**Authors:** Brian Kenny, Anthony Rosania, Helen Lu

**Affiliations:** 1 Emergency Medicine, Rutgers University New Jersey Medical School, Newark, USA

**Keywords:** clinical operations, emergency medicine barriers, emergency medicine physician, fast track, throughput

## Abstract

Objective*: *Lean methodology can be utilized to increase throughput in a fast-track care area without changing staffing in a busy, urban emergency department (ED).

Methods*: *A retrospective before-and-after analysis was performed to an improvement process in a fast-track care area within an ED with a census of 100,000 patients. The intervention utilized Lean methodologies to identify inefficiencies in the throughput model set for patients triaged to fast-track. Multiple ED stakeholders were involved in formulating a more efficient framework for how patients would receive care when triaged to fast-track.

Results: There was a decline in the patient's overall length of stay (-9%, p=0.08), arrival to the room (-10%, p=0.4418), and ED attending to disposition (-9%, p=0.003). Additionally, all aspects of patients leaving prior to treatment completion (against medical advice (-29.4%, p=0.006), elopement (-20.4%, p=0.049), and left without being seen (-5.3%, p=0.11)) declined.

Discussion*: *Identifying wasted time and resources in a patient's stay in the ED allowed for a more efficient throughput model to be developed. This resulted in patients being able to be seen in a more methodical manner leading to decreased wait times and lower left without being seen rates.

Conclusion: A Lean-based throughput model was implemented to improve efficiency, reducing the length of stay and increasing the volume of patients evaluated per shift without additional costs. This improvement led to fewer patients leaving before treatment and demonstrated the value of process improvement in healthcare.

## Introduction

Following a lull from the height of the COVID-19 pandemic, patient utilization of emergency departments (EDs) has rebounded, placing significant strain on hospitals to appropriately evaluate and treat an overwhelming number of patients [1]. Although the COVID-19 pandemic has ended, the residual effects of workforce shortages and limited hospital beds have exacerbated boarding issues [2]. ED overcrowding is associated with an array of poor clinical outcomes, including increased morbidity and mortality, compromised patient safety, decreased compliance with established guidelines, provider errors, patients leaving before treatment completion, increased healthcare worker burnout, and reduced patient satisfaction [3,4].

A proposed method to decrease overcrowding is to increase ED throughput for low-acuity patients [5]. An ED fast-track (FT) is typically designed to evaluate and provide a disposition for these lower-acuity patients quickly. Lean and Six Sigma methodologies can be utilized to identify errors and inefficiencies to increase both patient safety and throughput. Dr. Benjamin White's 2014 paper alluded to these concepts; however, there has been a lack of generalization of his findings throughout all health systems [5].

System improvement and change management in the ED have the potential to not only increase efficiency in patient care but also ultimately lead to improved quality of care [6]. By applying a systematic approach to the delivery of healthcare, variability declines, and quality increases. In concordance with improved efficiency, the Centers for Medicare and Medicaid Services (CMS) has included ED length of stay (LOS) and time from patient arrival to being seen by a physician in its list of publicly reported quality metrics [7].

This study focused on using a Lean-based approach to improve the efficiency of the FT care area in the ED, by restructuring available resources to optimize throughput without adding additional expenses. During a review of the current state of our department, there were numerous aspects during a patient's stay that had limited forward progress occurring in their care. Being able to identify aspects of inefficiency, which has been the foundational theory of Lean methodology, would be beneficial to increase the throughput in our FT care area. This process improvement strategy has been shown to work in various settings, including Nicklaus Children's Hospital (reducing code card turnaround time from three hours to 10 minutes through visual inspection and reducing excess supplies) and ThedaCare (equipping patient rooms with proper documentation equipment for nursing staff reducing the time nurses spent caring for each patient by 70%) [8]. These examples, in conjunction with Dr. White's publication, highlight that removing excess movement with limited progression behind those events helps to increase patient throughput.

## Materials and methods

Study design

This is a retrospective before-and-after analysis of an FT performance improvement process. Performance measures from X (pre) were compared to Y (post) over the previous 12-month period (September 2022-August 2023). The intervention occurred during the third and fourth quarters of 2023 and was completed by December 2023. This study focused on the throughput operations rather than individual patient records and therefore was exempted from a full institutional review board review.

Study setting and population

This process improvement was conducted in the large, academic, urban-based ED of the University Hospital in Newark, New Jersey, with an annual census of approximately 100,000. The hospital is an independently owned, 529-bed, tertiary care, and referral center. The overall ED admission rate is about 13%, with an additional 2.9% of patients in observation status. Patients are triaged through a standard process and directed to one of our ED's six care spaces based on patient age and acuity. The admission rate from our FT is 2.2% of those triaged to the care space, indicating that, despite these patients traditionally being of lower acuity, some of the patients triaged to the space require a higher level of care.

Selection of participants

We utilized data from the electronic health record from the previous 12 months to establish baseline characteristics of the patient care area. We included all patients triaged in the FT care area in the post-intervention period and cross-referenced with nursing and physician charting to ensure all patients were accounted for. Identifiable information was removed from data processing to ensure no breach of healthcare ethics occurred and data focused on throughput rather than patient characteristics. 

Data collection and processing

We collected data from an electronic health record system using its data analysis tool pack (i.e., SlicerDicer) during the pre- and post-intervention periods. Data points collected included LOS, disposition, acuity level, number of patients, and number of diagnostic imaging and lab orders.

Intervention

The improvement process utilized a Lean-based approach to identify inefficiencies in the throughput process in our FT [6]. The Lean methodology focuses on removing waste from a process and increasing the forward flow of progress. 

A committee was established consisting of ED physicians and nurses, ED technician staff, nurse managers, and a Lean and Six Sigma expert to engineer a plan for the future state of the care area. This planning included Lean and Six Sigma ideology to identify points of variation and to forecast conflicts that could arise. The goal was to reduce non-clinical time in exam rooms by having continued forward progress present in a patient's care plan. A value stream mapping was created to help identify areas of the process that could be improved. 

Patients were directed to the FT care space after triage; this aspect of the throughput flow was not altered. During the pre-intervention period, patients triaged to the FT area would wait for room availability and remain in the room until a disposition was completed.

During the intervention phase, patients would be roomed in the care space and evaluated simultaneously by a registered nurse (RN) and either a nurse practitioner (NP) or physician. After evaluation, patients would receive further care in an adjacent procedure room as needed (e.g., IV placement or laceration repair) or directed to the FT results waiting area. Patients would receive their disposition from this waiting area or be brought back into a procedure exam room if needed for reevaluation and consultation.

The initial improvement trial used physicians and NPs involved in the planning process. Subsequent trial sessions were with clinicians who were asked and agreed to participate in the study (trial 2), followed by a period of clinicians who were told the day of their shift of the change in throughput for the day (trial 3).

Methods of measurement

The primary outcomes measured were FT LOS (defined as the time interval between patient registration in the ED and patient departing the ED), median arrival to the room, first attending to disposition, and percentage of patients leaving before treatment completion in the entire department. We utilized the Mann-Whitney U testing for non-normalized data to assess the significance of the LOS change and first attending to disposition (given that these measurements are variable and are found to be not normally distributed). T-tests were used to determine if there was a difference in the average number of patients leaving before treatment completion (given this data was found to be normally distributed). The acuity level and percentage of patients requiring labs, CT, X-ray, and ultrasound remained similar during the study periods.

## Results

Table 1 and Table 2 highlight the data that was collected during the initial pilot phases of this improvement project. Our ED evaluates on average 51 patients in the FT care area per day, with 2.2% being admitted and 1.3% in observation status. Peak arrival times to the FT care area are between 7 AM and 4 PM, with a height of 5-6 patients per hour during this time. During the baseline data collection period, 20,093 patients were triaged to FT. Of the patients triaged to FT, 10,610 (52.8%) were Emergency Severity Index (ESI) level 3, 8,569 (42.6%) were ESI level 4, and 410 (2%) were ESI level 5 (ESI triages patients based on acuity with 1 being most acute and 5 being the least acute). Of all these patients, all received an exam, 8,298 (41.3%) had laboratory studies obtained, 3,195 (15.9%) had a CT scan ordered, 5,666 (28.2%) had an X-ray, and 241 (12%) had an ultrasound.

**Table 1:** Patient characteristics during the baseline preceding 12 months and during the three trial phases of the process. Patient characteristics throughout the trials in the fast-track pilot process. There were a total of 20,093 patients triaged to fast-track during the baseline period, 60 patients in trial 1, 59 patients in trial 2, and 60 patients in trial 3. Each group had a similar percentage of patients with comparable levels of ESI-rated acuity that required diagnostic studies and subsequent admission owing to the groups' overall similarity. ESI: Emergency Severity Index

Patient characteristics	Baseline data (previous 12 months)	Trial 1	Trial 2	Trial 3
ESI 1	1 (0.005%)	0 (0%)	0 (0%)	0 (0%)
ESI 2	380 (1.9%)	0 (0%)	0 (0%)	0 (0%)
ESI 3	10,610 (52.8%)	35 (58.3%)	26 (44.1%)	29 (48.3%)
ESI 4	8,569 (42.6%)	24 (40%)	28 (47.5%)	27 (45%)
ESI 5	410 (2%)	1 (1.7%)	4 (6.8%)	3 (5%)
Patients with labs	8,298 (41.3%)	26 (43.7%)	25 (42.3%)	22 (36.6%)
Patients with CT	3,195 (15.9%)	7 (11.3%)	7 (11.9%)	7 (11.6%)
Patients with X-ray	5,666 (28.2%)	15 (25.4%)	14 (23.7%)	14 (23.4%)
Patients with ultrasound	241 (12%)	13 (21.1%)	3 (5%)	7 (11.6%)
Patients admitted	442 (2.2%)	3 (5%)	1 (1.6%)	3 (3.3%)
Patients observed	261 (1.3%)	1 (1.6%)	0 (0%)	0 (0%)

**Table 2:** Patient-centered metrics evaluated throughout the trial period. A p-value of <0.05 was considered significant. The total number of patients in the trial period was 20,093; the combined patient volume during the trial period was 179. The time for first attending to disposition, patients seen during an eight-hour period (this time was used as a marker for average patients seen as this is the standard length of a shift in this care area), and percentage of patients who eloped or left against medical advice were all found to be statistically significant. There was no significant difference in metrics between patients seen by physicians or nurse practitioners. Elope: elopement, leaving the emergency department after treatment is initiated without informing staff; AMA: against medical advice, leaving the emergency department prior to treatment completion after informing staff; LWOB: left without being seen, leaving the emergency department prior to evaluation by a provider

Metric	Baseline	Trial data	Percent change
Medial arrival to room (min)	103	93	-10 (p=0.4418)
First attending to disposition (min)	178	62	-9 (p=0.003)*
% admitted	442 (2.2%)	7 (3.9%)	+77 (p=0.325)
% observed	261 (1.3%)	1 (0.5%)	-68 (p=0.224)
Elope % (department)	985 (4.9%)	7 (3.9%)	-20.4 (p=0.049)*
AMA % (department)	341 (1.7%)	2 (1.1%)	-29.4 (p=0.006)*
LWOB % (department)	1889 (9.4%)	16 (8.9%)	-5.3 (p=0.11)
Avg length of stay (min)	359	328	-9 (p=0.08)
Avg patients seen in fast-track per eight hours	17	20.5	21% (p=0.0001)*

Table 2 shows that the LOS declined by 9% and there were 21% more patients seen per shift during this trial. The three components of patients leaving before treatment completion (elopement, against medical advice, and leaving without being seen) all declined. Additionally, the Mann-Whitney testing showed a statistically significant change in attending to disposition with a p-value of <0.05. There was no statistically significant difference in average patient LOS; however, there was a statistically and clinically significant noted difference in the volume of patients seen in the care space. The LOS declining by 9% allowed for 21% more patients to be seen during a standard shift. T-test calculations for patients leaving against medical advice (p=0.006) and elopement (p=0.049) showed significance; however, patients leaving without being seen were shown to not have a statistically significant difference (p=0.11).

## Discussion

Our ED evaluates on average about 300 patients per day, with about 51 patients being triaged to FT per day. Our ED utilizes an ESI triage score of 1 (most acute) to 5 (least acute) based on a systematic algorithm based on projected needed resources, limiting potential bias. Historically, care spaces that were meant to serve as an efficient throughput space, such as an FT, would see mainly levels 4 and 5. In this urban, level 1 trauma center ED, 53% of patients presenting to the FT area were ESI level 3; however, when compared to level 3 patients triaged to the acute care area, the admission percentage was significantly lower. When triaged to the acute care area, level 3 patients had an admission rate of 17.4%, whereas FT was only 2.9%. This highlights that despite being an ESI level 3, patients were still requiring fewer services when going to FT.

In this single-center trial, a focused Lean-based throughput model was able to decrease LOS by 31 minutes and increase patients seen by 3.5 per shift without adding expense and with limited resource usage. These results have significant implications that could be widely adapted to help increase ED throughput and its subsequent implications. By decreasing the time that patients are in the room and decreasing patient LOS, more patients can access care which was evident by the decline in patients leaving before treatment is complete. This process has exemplified the value of using systems thinking and improvement science to change healthcare ideology and operations and improve patient throughput [7,8].

While the intervention required an effort in the planning and data collection phases, the operational and throughput benefits outweighed the resources that were utilized in creating this project. This was highlighted by the decrease in LOS and increase in patients seen per shift, without adding additional staffing to the care space. These extraordinary results are typical of systems engineering fixes, and when individuals buy into the Lean methodology approach, a cultural change often occurs as future project ideas develop after the realization of this method's success [9,10]. The ability to work in a team-oriented nature with physicians, nurses, and technicians all led to greater success and comradery with the patients ultimately benefiting the most from the project [11,12].

The approach was centered on decreasing the time to physician/NP, eliminating non-valued exam room time, effective communication on patient whereabouts, and planning for an influx of patients [13]. We utilized a concept to see new patients in six out of the 12 exam rooms, utilizing then the other six rooms and a set of chairs for patients requiring labs, procedures, or IV infusions and medications. After evaluation in the exam room patients were transitioned to a results waiting care space pending a disposition. Through this process, the LOS decreased by 31 minutes (9%), allowing an additional three patients per shift to be seen (an increase of 21%). Although not a statistically significant difference, there is clear clinical value to being able to evaluate patients more efficiently in the care space. Additionally, patients signing out beforehand and eloping from the department decreased significantly. Patients leaving prior to being seen remained stagnant as these patients were leaving prior to being triaged, a portion of the patient arrival throughput that was left unchanged in this process. Table 1 highlights that the baseline characteristics of the patients were similar, thus removing any potential bias to patients that would need fewer resources that could skew the data.

During this period of ever-increasing ED overcrowding and patient boarding, solutions like this project can be widely adapted to increase throughput and decrease LOS [13,14]. These benefits ultimately increase a department's efficiency and capacity, with limited associated resources added [15].

There are limitations to this study as it was conducted. As with any before-after studies, the causality is difficult to confirm, and the outcomes measured are a mere association. Additionally, this study was performed at a single institution, with a specific type of population, and thus, the generalizability of the process would be challenging in EDs with different demographics or without a designated FT area. Despite this, given that change management and improvement science can be broadly applied, our findings represent an opportunity of value for ED administrators regardless of the type of department [16]. Staff involved in the improvement process were unaware of specific data being collected but were unable to be blinded to the intervention due to the need to discuss the changes in patient throughput. Also, patients were not blinded to the intervention, which raises the concern for potential bias due to the Hawthorne effect. This intervention that was studied consisted of several small incremental systems improvements grouped into one large process change. While this is the typical case with process improvement and systems engineering, this limits the ability to interpret the results of each change [17]. The majority of patients in our department who leave prior to being seen do so from the main waiting room; thus, although the throughput in the FT area improved, if patients were triaged to another care area, the left without being seen rate would be unchanged. 

As the trial period has concluded, a significant barrier that has presented itself is the ability to have the process implemented consistently in the care space without specific instruction to use the new throughput model. Our future goal is to instill permanent change in FT to make the transition for physicians, NPs, and nurses who are accustomed to the original method more seamless and easier to adapt [18-22]. Despite using the Lean methodology to identify aspects that could be improved on, there is still the change management and sustainability of change for process improvement projects which is a significant limiting factor to the success of an intervention such as this one. Utilizing a similar process by involving all potential stakeholders early in a process improvement is a key initial step for a project's success. Including various perspectives on a department's throughput model allows all potential inefficiencies and wasted time in a patient's stay to be identified.

## Conclusions

A Lean-based throughput model was implemented to improve efficiency, reducing the LOS and increasing the volume of patients evaluated per shift without additional costs. This improvement led to fewer patients leaving before treatment and demonstrated the value of process improvement in healthcare. 

Despite the successful outcomes, limitations include the study's single-center focus and challenges in generalizing results to other EDs. The intervention's effectiveness could be influenced by factors such as staff awareness and patient characteristics. Future efforts aim to embed the new model permanently into FT operations to ensure consistent application and adaptation by medical staff. There is a need for further research to apply this model in other diverse settings to confirm generalizability and applicability to other sectors of the healthcare system. 
